# Interpreting Signal Amplitudes in Surface Electromyography Studies in Sport and Rehabilitation Sciences

**DOI:** 10.3389/fphys.2017.00985

**Published:** 2018-01-04

**Authors:** Andrew D. Vigotsky, Israel Halperin, Gregory J. Lehman, Gabriel S. Trajano, Taian M. Vieira

**Affiliations:** ^1^Department of Biomedical Engineering, Northwestern University, Evanston, IL, United States; ^2^Physiology Discipline, Australian Institute of Sport, Canberra, ACT, Australia; ^3^Centre for Exercise and Sport Science Research, School of Medical and Health Sciences, Edith Cowan University, Joondalup, WA, Australia; ^4^Private Practice, Toronto, ON, Canada; ^5^School of Exercise and Nutrition Sciences, Queensland University of Technology, Brisbane, QLD, Australia; ^6^Institute of Health and Biomedical Innovation, Queensland University of Technology, Brisbane, QLD, Australia; ^7^Laboratory for Engineering of the Neuromuscular System, Department of Electronics and Telecommunications, Politecnico di Torino, Turin, Italy

**Keywords:** strength, hypertrophy, rate coding, motor unit recruitment, exercise, activation, excitation, muscle force

## Abstract

Surface electromyography (sEMG) is a popular research tool in sport and rehabilitation sciences. Common study designs include the comparison of sEMG amplitudes collected from different muscles as participants perform various exercises and techniques under different loads. Based on such comparisons, researchers attempt to draw conclusions concerning the neuro- and electrophysiological underpinning of force production and hypothesize about possible longitudinal adaptations, such as strength and hypertrophy. However, such conclusions are frequently unsubstantiated and unwarranted. Hence, the goal of this review is to discuss what can and cannot be inferred from comparative research designs as it pertains to both the acute and longitudinal outcomes. General methodological recommendations are made, gaps in the literature are identified, and lines for future research to help improve the applicability of sEMG are suggested.

## Introduction

Surface electromyography (electromyography, EMG; surface EMG, sEMG) is a common research tool used to investigate a wide range of research questions across various disciplines. sEMG is perhaps most useful for providing insight into how the neuromuscular system behaves. In its simplest sense, sEMG is a highly-sensitive voltmeter that detects depolarizations and hyperpolarizations (increases and decreases in voltage, respectively) that occur on the sarcolemma (muscle fiber membrane). These depolarizations are necessary for, and precede, the contraction of a muscle. Among other disciplines, the utilization of sEMG is popular in sport and rehabilitation sciences, with the number of studies including the terms “EMG” and “exercise” increasing exponentially since 1950 (*R*^2^ = 0.98) (Corlan, 2004). In those studies, sEMG amplitudes collected from different muscles are commonly compared between exercises, techniques, and/or loads. Based on such comparisons, researchers often attempt to draw conclusions that can be separated into two primary categories: (1) inferring mechanisms and (2) predicting longitudinal outcomes. Included in acute, mechanistic variables are activation, force production and sharing characteristics, and recruitment strategies. Knowledge of such mechanistic variables is expected to inform researchers and practitioners as to the underpinning of why unique adaptions occur and through which pathways. Conversely, inferred longitudinal outcomes primarily consist of muscle hypertrophy and increases in strength, in that it is assumed that greater sEMG amplitudes are predictive of greater adaptation (Andersen et al., 2006; Escamilla et al., 2010; Reiman et al., 2012; Tsaklis et al., 2015; Halperin et al., 2017). Oftentimes, however, such conclusions are unsubstantiated and unwarranted. These misinterpretations mainly stem from (1) the complicated nature of sEMG and (2) lack of longitudinal work. This leaves both researchers and practitioners with the important question: If we cannot make such conclusions, what does sEMG tell us in the context of exercise?

Numerous pieces have been written on the shortcomings of sEMG in the exercise fields (Cavanagh, 1974; De Luca, 1997; Farina, 2006; Enoka and Duchateau, 2015). Indeed, influential biomechanists have been writing about similar issues since the 1970s (Cavanagh, 1974). However, in our view, few have attempted to simplify the neuro- and electrophysiological concepts in their discussion, making it difficult for those who lack a strong relevant background to fully understand the content. Thus, our goal with this review is to allow the applied exercise scientists and educated coaches and practitioners to develop a better understanding of this important topic. Further, rather than a general overview of sEMG that pertains to many research designs and fields, we aimed to narrow our discussion to a number of common research designs in the exercise domain. With this in mind, the purpose of this review is manifold. First, we discuss what conclusions cannot be drawn from sEMG studies in sport and rehabilitation sciences. This subsection will cover both acute and longitudinal inferences. Next, we will discuss what conclusions can be drawn from sEMG studies and what experimental considerations need to be made before such conclusions can be drawn. Lastly, we will identify gaps in the literature and suggest lines for future research to help improve the applicability of sEMG.

### Terms and definitions

**Figure 1:**
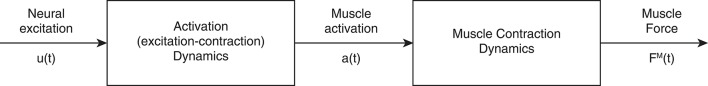
Production of muscle force from neural input. Excitation is the electrochemical input from the central nervous system into the muscle. This signal triggers excitation-contract coupling, which leads to an active muscle state (activation). Finally, muscle force is produced after cross-bridges are formed and force is transmitted through the muscle. Adapted from Zajac (1989).

Despite the apparent simplicity of sEMG, many terms relating to EMG and neuromuscular physiology are often misused and conflated with one another. Therefore, before delving into the primary topics of this review, it is necessary to operationally define a number of technical terms relating to EMG and neuromuscular physiology. A schematic of how many of these definitions relate to one another can be found in Figure 1.

- **Neural Excitation**–Electrochemical input from an α-motoneuron that depolarizes all of the muscle fibers that it innervates (Gottlieb et al., 1990; Winters, 1990; Zatsiorsky and Prilutsky, 2012).
- **Muscle Excitation**–Depolarization of the sarcolemma following neural excitation, delivered to the muscle via the neuromuscular junction (Farina et al., 2016). This is also referred to as the *muscle fiber action potential*.
- **Activation Dynamics**–Events following excitation that cause a muscle to produce active force via actin-myosin cross-bridging, also known as excitation-contraction coupling (Gottlieb et al., 1990; Winters, 1990; Zatsiorsky and Prilutsky, 2012). These events are affected by the state of the muscle (i.e., previous activation), and thus, so are the events that follow (Figure 1).
- **Muscle Activation**–The active state of a muscle, ranging from 0% (all fibers inactive) to 100% (all fibers active) (Gottlieb et al., 1990; Winters, 1990; Zatsiorsky and Prilutsky, 2012).
- **Muscle Contraction Dynamics**–Takes into account the length and velocity of the muscle (Zajac, 1989). Many phenomenological models exist to represent these dynamics, such as the Hill muscle model (Hill, 1938), the Huxley cross-bridge model (Huxley, 1957), and the three-filament model (includes titin) (Herzog et al., 2012, 2015).
- **Muscle Force**–Force that is produced by the muscle.
- **Surface EMG**–Electrophysiological recording technology used for the non-invasive detection of the electric potential resulting from the transmembrane current of muscle fibers (muscle excitation). With appropriate processing and based on sufficiently reasonable approximations, sEMG may provide information on the timing and degree of muscles' excitation (Zajac, 1989)^1^.

## What conclusions *cannot* be drawn from sEMG studies?

### Acute and mechanistic variables

In this section, we cover a number of acute and mechanistic variables that are commonly collected, analyzed, and interpreted with the goal of developing a better understanding of force production and the pathways leading to force generation. Specifically, motor unit recruitment, rate coding, activation, force generation, and force sharing are discussed in view of the common interpretation they receive in the sport and rehabilitation sciences.

#### Motor unit recruitment and rate coding

**Figure 2:**
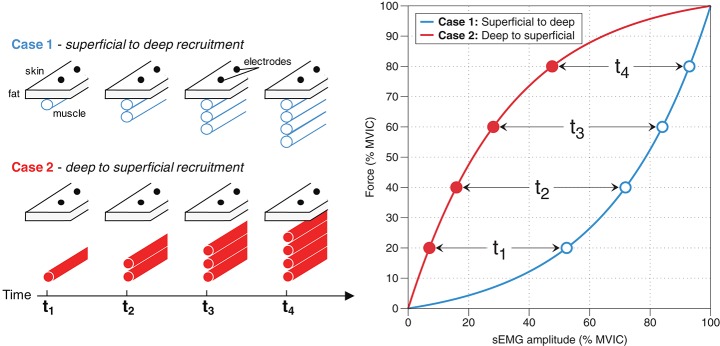
Recruitment methods and their effects on sEMG amplitude. Case 1: If a muscle recruits motor units from superficial to deep, then this will result in sEMG amplitude rising at a faster rate than force; that is, sEMG amplitude (% MVIC) ≥ Force (% MVIC). Case 2: If a muscle recruits motor units from deep to superficial, then this will result in force levels rising at a greater rate than sEMG amplitude; that is, Force (% MVIC) ≥ sEMG amplitude (% MVIC).

A motor unit is the fundamental unit that converts efferent action potentials to force. It consists of a motor neuron and all of the muscle fibers that said motor neuron innervates. There are two primary mechanisms by which the nervous system enable a muscle to produce more force^2^: (1) motor unit recruitment, in which more motor units are utilized, and (2) rate coding, in which the motor units that are already recruited “fire” at a faster rate (Kukulka and Clamann, 1981; van Bolhuis et al., 1997; Farina et al., 2016). Importantly, the relative contribution of motor unit recruitment and rate coding depends on the muscle group (Kukulka and Clamann, 1981; De Luca et al., 1982; De Luca and Kline, 2012). To complicate things further, these recruitment characteristics are task-dependent, in that they change with different rates of force development, like during ballistic contractions, and thus are not specific to force levels *per se* (Desmedt and Godaux, 1977). Unless the specific recruitment characteristics of a muscle are known, one cannot discern motor unit recruitment from rate coding using sEMG amplitude. Moreover, *how* a muscle recruits its motor units will greatly affect sEMG amplitude; for example, a muscle that recruits motor units from deep to superficial will display a different sEMG amplitude-force relationship than would a muscle that recruits motor units from superficial to deep, assuming rate coding characteristics are equal (Figure 2; Farina et al., 2016). This is exemplified by Mesin et al. (2010), who found that the tibialis anterior likely has a superficial-to-deep recruitment pattern, and thus has an sEMG amplitude-force relationship similar to that depicted in Case 1 in Figure 2. Whether there is a preferential recruitment direction for different muscles or circumstances has yet to be determined. It is clear that heterogeneity in motor unit recruitment and rate coding patterns precludes one from making conclusions about either characteristic from sEMG amplitude alone.

Although there are methods to investigate motor unit recruitment and rate coding using sEMG—such as spike-triggered averaging and spectral analyses—these techniques are complex and their validity, especially during dynamic, high-intensity, or fatiguing exercise, is questionable (Farina et al., 2010, 2014). Moreover, sEMG amplitude alone, which is likely the variable most-often reported from sEMG experiments, cannot be used to infer motor unit recruitment or rate coding. This is because both motor unit recruitment *and* rate coding have significant, and nearly equal, contributions to time-averaged sEMG amplitude [root mean square (RMS) or average rectified value (ARV)] under non-fatiguing conditions (Farina et al., 2016). Hence, since the relative contribution of each of the two pathways cannot be separated using sEMG amplitude, the conclusions that can be drawn for each mechanism are limited. Indeed, a recent model suggests that similar recruitment can occur under different loads with different levels of excitation, simply due to differences in rate coding (Potvin and Fuglevand, 2017). In addition, under fatiguing conditions and following training periods, sEMG amplitudes may be altered by intra- and extracellular ion concentrations and motor unit synchronization in unintuitive ways (Dimitrova and Dimitrov, 2003; Arabadzhiev et al., 2010a,b). These alterations affect sEMG amplitudes and confound the neural drive collected with sEMG with other measures (Arabadzhiev et al., 2010a,b; Dideriksen et al., 2011). From these inherent characteristics, it is clear that the array of contributors to sEMG amplitude precludes one from drawing conclusions that pertain to motor unit recruitment and rate coding from sEMG amplitude. Hopefully, future work implementing new, more sophisticated technologies, like high-density (HD) EMG and decomposition techniques, may allow for greater insight into these mechanisms (Merletti et al., 2008). However, until such technologies are available and validated, researchers are limited as to the mechanistic conclusions that can be drawn from sEMG signals (Farina et al., 2010, 2014; Del Vecchio et al., 2017).

#### Activation

**Figure 3:**
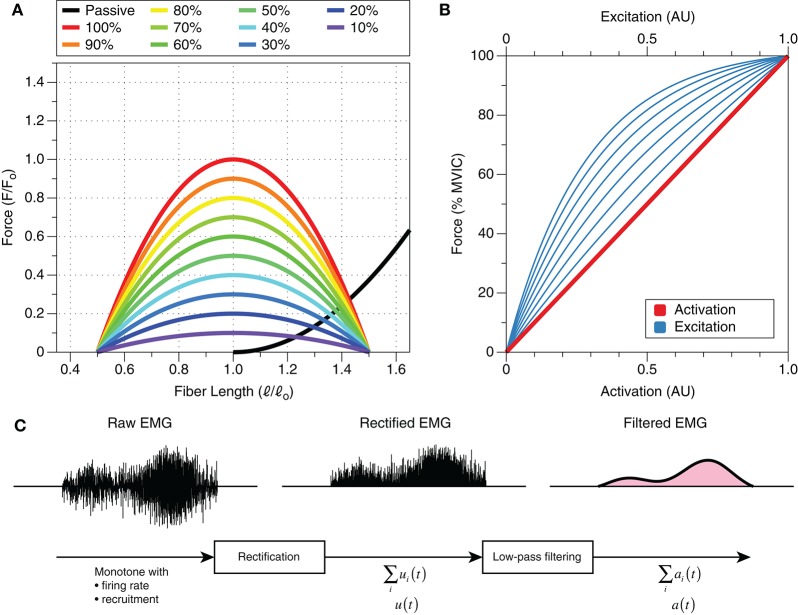
The isometric relationships between muscle force, activation, excitation, and fiber length. **(A)** Only the active curve is affected by activation, and any activation can occur regardless of the muscle's normalized force output. That is, activation is independent of fiber length; normalized force is a function of fiber length *and* activation, in addition to contraction velocity (not shown). The passive length-tension curve is unaffected by activation; this has important implications for force production and force sharing. Both force and length are represented relative to force and length, respectively, at optimal length^a^. **(B)** Excitation is curvilinearly related to activation and force, whereas force and activation are directly related to one another (one-to-one). Different muscles have different excitation-activation relationships, and thus, excitation for a given muscle can be one of the numerous lines that are plotted. Graph derived from Potvin et al. (1996) and Lloyd and Besier (2003). **(C)** Raw EMG is influenced primarily by motor unit recruitment and rate coding. The envelope of this signal, rectified EMG, may be considered the sum of neural drive to the area of muscle over which the electrode is placed, which, *somehow* [e.g., Σu_i_(t)], is related to excitation, u(t). By filtering this signal, one can obtain data that are related to activation, a(t). Adapted from Zajac (1989). ^a^The optimal length shifts to the *right* with decreasing levels of activation (Lloyd and Besier, 2003; de Brito Fontana and Herzog, 2016), but a thorough description of these changes is outside the scope of this review.

Muscle activation refers to the state of the muscle and is related to the magnitude of force that a muscle actively produces (i.e., not including passive contributions) relative to its maximum ability to produce force actively (Figure 3A). Although force is related to activation, force and activation differ in a number of ways. First, activation only deals with active contributions to muscle force and thus ignores passive components (Figure 3A). Second, activation is essentially a scaling factor, which relates active force and maximum potential active force at a given fiber length and velocity (Zajac, 1989; Figure 3B). Lastly, activation is unaffected by length and velocity, whereas force production is highly influenced by fiber length and velocity. This is because activation does not take into account muscle contraction dynamics (force-length, force-velocity, history dependence, etc.). In other words, activation is related to the number of fibers that are active and not the force-generating capacity of those fibers.

Rather than measuring activation, sEMG measures changes in the polarity of the muscle fibers' membrane resulting from *neural excitation*; in other words, sEMG is a measure of *muscle excitation*. Activation and excitation differ in that excitation is a precursor to activation (Figure 1). Specifically, activation takes into account excitation-contraction dynamics (Zajac, 1989), including electromechanical delays, ion kinetics, etc., whereas excitation does not (Figure 1). Activation can be estimated from muscle excitation and sEMG, but the relationship is not straightforward (Figures 3B,C; Lloyd and Besier, 2003; Staudenmann et al., 2010). Because sEMG is not a direct measure of muscle activation, we suggest that authors avoid using the term “activation” when referring to sEMG amplitudes. Instead, authors should simply utilize the term “sEMG amplitude,” “muscle excitation,” or “myoelectric activity.” Moreover, we also propose that the term “muscle activity” is ambiguous and should be avoided, as muscles can provide large contributions to a movement without being excited.

#### Force production and sharing

sEMG is particularly attractive because its primary constituents, motor unit recruitment and rate coding, are also the precursors to active force generation (Staudenmann et al., 2010). As such, one may be eager to associate sEMG amplitudes, especially those that are normalized to maximum voluntary isometric contractions (MVIC), with muscle force. This attraction has been eloquently and satirically described by Dr. Peter Cavanagh in 1974, “The day that most electromyographic kinesiologists are collectively awaiting, with the enthusiasm of Doomsday watchers, is that when they can use some measurement derived from the electromyogram to indicate the force being produced by a muscle group during unrestricted movement of the body” (Cavanagh, 1974). Just like in 1974, doing so today is erroneous for a number of reasons. First, the passive force-length curve is ignored (i.e., force contributions from non-contractile structures, such as collagen/extracellular matrix, tendons, fascia, titin, etc.). A muscle can produce force with an sEMG amplitude of zero due to its passive properties (Figure 3A–black line). Second, the active length-tension curve and contraction history of a given muscle must be taken into account (Herzog et al., 2015). The often-cited curvilinear relationship between sEMG amplitude and force pertains to isometric contractions at one joint angle. However, when muscles change length, or even have velocities, then this relationship becomes much more complicated (Farina, 2006). When considering the active length-tension relationship, an activation of 70% at a muscle length of 0.8^3^ will produce similar force to an activation of 60% at a relative fiber length of 1.0 (Figure 3A). Contraction history, on the other hand, tells us that a muscle can produce more force following an eccentric contraction than a concentric one (Herzog, 2004; Herzog et al., 2015; Seiberl et al., 2015). Lastly, the amplitude of sEMG detected locally from the muscle does not take muscle properties into account—that is, physiological cross-sectional area and normalized muscle force—which will affect the force-generating capabilities of muscle (Zajac, 1989). These cautionary points have been elucidated *in vivo*, in animal work that has shown that EMG cannot reliably predict muscle force during dynamic tasks (Roberts and Gabaldón, 2008). The inherent heterogeneity and dynamic nature of the neuromuscular system preclude the deduction of muscle force from normalized sEMG signals.

Force sharing is highly related to force production, as it aims to understand how different muscles contribute to a net joint moment. It is quite logical to think that sEMG would provide valuable insight for force sharing, but not only do the aforementioned points on force production apply, but differences in muscle architecture must also be considered (e.g., physiological cross-sectional area, muscle moment arms, etc.). To estimate force sharing, one must utilize musculoskeletal modeling rather than *just* sEMG; however, sEMG can be quite useful for “informing” musculoskeletal models (Sartori et al., 2016). Such models are enormously complex, but they are necessary to better describe force sharing characteristics. Without such models, sEMG amplitude alone does not provide great insight into force sharing characteristics of mechanically redundant muscles.

#### Interim summary

Collectively, sEMG studies that aim to draw conclusions about muscle force production, muscle activation, or mechanisms of force production are problematic when based solely on sEMG amplitude. Oftentimes, in order to draw mechanistic conclusions, more advanced sEMG processing and modeling techniques are needed. At face value, at best, sEMG amplitude is strictly indicative of muscle excitation.

### Longitudinal outcomes

Longitudinal outcomes are undoubtedly the most applicable, clinically relevant measures that a sport or rehabilitation scientist can provide to a practitioner. In this section, we discuss two commonly-measured and sought after outcomes: muscular strength and hypertrophy. Measuring these outcomes, however, can be quite difficult and time-consuming (Halperin et al., 2017). Before their discussion, it is important to introduce the term “surrogate endpoint,” borrowed from the medical literature. Surrogate endpoints are often utilized to assess a difficult-to-measure variable—such as one that may take months or years to occur (like strength or hypertrophy)—with greater ease (Halperin et al., 2017). For example, cholesterol and blood pressure have been used as surrogate endpoints for cardiovascular health (Fleming and DeMets, 1996). Implied by the idea of a surrogate endpoint is that, not only is the surrogate endpoint correlated with the clinical endpoint or real variable of interest, but net effects of interventions on clinical outcomes are also reflected (Prentice, 1989; Fleming and DeMets, 1996). A myriad of studies has attempted to utilize sEMG as a surrogate endpoint of both strength and hypertrophy (Andersen et al., 2006; Escamilla et al., 2010; Reiman et al., 2012; Tsaklis et al., 2015; Halperin et al., 2017). While such studies may provide insight into the neuromuscular system and how changes in exercise variables, ranging from the exercise itself to load, may affect muscle excitation, at present, it is unknown if greater muscle excitation measured with sEMG is predictive of long-term adaptation, as studies investigating the predictive validity of sEMG are lacking (Halperin et al., 2017).

#### Hypertrophy

Muscle hypertrophy, or the growth of muscle fibers, is a highly sought-after exercise outcome for individuals ranging from patients with sarcopenia to physique competitors. Due to its longitudinal nature, it can be quite difficult and time-consuming to measure experimentally. As such, sport and rehabilitation scientists have commonly utilized sEMG to acutely compare exercises and loading schemes to help drive exercise programming. However, recommendations from these studies are often unjustified and ill-advised as, at present, there are no longitudinal studies to suggest that sEMG is predictive of hypertrophic outcomes (Halperin et al., 2017).

The premise upon which the assumption that sEMG amplitude may be useful for predicting hypertrophy could be challenged on a theoretical level; that is, muscle excitation is predictive of hypertrophic outcomes. A number of avenues have been explored that may challenge this premise, including the relationship between muscle protein synthesis (MPS) and hypertrophy, the presence of hypertrophy without muscle excitation, and differential hypertrophy with similar excitation, which are explained herein.

Acutely, it has been postulated that maximum motor unit recruitment, a primary constituent of the sEMG signal, is important for stimulating hypertrophy (Wernbom et al., 2007; Marcotte et al., 2015; Dankel et al., 2017). From a more mechanistic standpoint, the presumed implicit (NB this has not been explicitly stated) rationale for sEMG amplitude being able to predict hypertrophy would be that greater sEMG amplitude implies greater muscle excitation, which implies greater MPS (Kumar et al., 2009; Holm et al., 2010), which implies a greater hypertrophic response. This argument breaks down when considering that acute fractional protein synthesis, a measure of MPS, is not correlated with hypertrophy (Mitchell et al., 2014). Thus, even if sEMG amplitude was a valid surrogate for MPS, a study that to our knowledge has yet to take place, it will not necessarily be predictive of hypertrophic outcomes.

It has been known since the 1970s that a muscle does not need to be excited for growth to occur (Goldberg et al., 1975). In fact, a hypertrophic response can be elicited in denervated muscle by applying mechanical tension (stretch) (Sola et al., 1973). Such findings, that stretch itself is a stimulus for hypertrophy, were recently replicated in humans, in a study that demonstrated an increase in gastrocnemii muscle thickness following 6 weeks of static stretching (Simpson et al., 2017). Taken together, it is clear that a muscle does not *need* to be excited to grow; however, this evidence does not suggest that hypertrophy can be *maximized* without a muscle being excited.

More recent data further support the idea that muscle excitation alone cannot predict hypertrophy: Eftestøl et al. (2016) divided 20 rats into two groups: 10 high-load (100% of maximum isometric strength) and 10 low-load (50–60% of maximum isometric strength, thus resulting in a fast, concentric contraction). Experimenters stimulated tibialis anterior (TA) and extensor digitorum longus (EDL) with identical electrical stimulation every other day for 6 weeks (Eftestøl et al., 2016). It was found that the magnitude of hypertrophy of both TA and EDL were dependent upon and proportional to the load (Eftestøl et al., 2016). Therefore, hypertrophic responses were dependent on more than just excitation. Of relevance, activation-independent (not excitation-independent) differences in hypertrophy have also been shown to occur in humans. Noorkoiv et al. (2014) trained young adult males using isometric knee extensions at short *or* long muscle lengths (ascending and descending limbs of the length-tension curve, respectively), with loads corresponding to 80% of each participant's MVIC strength, measured at each participant's optimal joint angle, over a period of 6 weeks. Only participants who trained at long muscle lengths experienced increases in quadriceps muscle volume (Noorkoiv et al., 2014). From these data, it can be argued that hypertrophic responses were either independent of activation or, alternatively, greater with what may have been slightly lower activation, as passive-elastic forces may have contributed to the long-length condition (Noorkoiv et al., 2014). One may argue that the relationship between force output and sEMG amplitude is suggestive that sEMG may be able to serve as proxy for the force “felt” by a given muscle, and thus hypertrophy, but this relationship is muscle-length specific and can become quite complex, as discussed in the preceding section (section Acute and Mechanistic Variables).

In addition to the above, substantial evidence suggests that there is a disconnect between sEMG amplitudes and hypertrophy during highly-fatiguing conditions. Mitchell et al. (2012) measured the effects of 3 sets of 30% vs. 3 sets of 80% vs. 1 set of 80% of one-repetition maximum (1 RM) knee extensions on quadriceps muscle hypertrophy. Investigators found that 3 sets of 30% and 3 sets of 80% elicited similar growth (Mitchell et al., 2012). In an attempt to elucidate the mechanisms underlying these outcomes, Jenkins et al. (2015) carried out a sEMG study and found that, when taken to momentary muscular failure, 3 sets of 80% 1 RM elicited greater sEMG amplitude than did 3 sets of 30% 1 RM. These results indicate that sEMG amplitude alone cannot be used to predict hypertrophic outcomes in highly-fatiguing conditions (Vigotsky et al., 2016) and are consistent with several other studies, which have investigated either hypertrophy or sEMG (Schoenfeld et al., 2014, 2016, 2017; Looney et al., 2016).

Both basic science and applied research challenge the notion that excitation—the construct measured by sEMG—is necessarily predictive of hypertrophy, as other variables influence both sEMG amplitude and the strength and hypertrophy responses to resistance training. Notwithstanding these data, complementary data are necessary to paint a broader and more complete picture of the potential role of excitation in hypertrophy. Specifically, results from experiments in which excitation is the independent variable and load is a control variable are needed. For example, a study in which the load utilized is equal to 50% MVIC and excitations are 50% (isometric) and 90% (concentric) of maximum. A related study could explore the influence of sEMG amplitude on hypertrophy while keeping constant muscle length and contraction velocity. Moreover, it is important to note that absence of evidence is not evidence of absence; although sEMG has not been established to be predictive of hypertrophy, it does not mean that no relationship exists. Some indirect evidence loosely suggests that sEMG amplitude may have a potential role in predicting hypertrophy: When comparing rectus femoris sEMG amplitude in single-joint exercises (knee extension) to that elicited by multi-joint exercises (simultaneous hip and knee extension), it is clear that the former elicits much greater sEMG amplitudes (Yamashita, 1988; Ema et al., 2016). Separate, longitudinal evidence suggests that single-joint exercises are effective for rectus femoris hypertrophy (Ema et al., 2013), while multi-joint exercises (barbell back squats) are not (Fonseca et al., 2014; Earp et al., 2015). These data alone are not enough to suggest that a strong, predictive relationship exists, especially because the data are from different cohorts, but taken together may be used as a rationale to investigate whether a relationship does indeed exist. In other words, using these data to justify a relationship is tautological and lacks pragmatism; put much more eloquently, “… one must not verify an idea using the same data that suggested the idea in the first place” (Feynman et al., 2011).

#### Strength

Strength, like hypertrophy, is an outcome of resistance training that is highly desirable. Because there are many ways to test strength (Buckner et al., 2016), the working definition of strength within the context of this piece is the ability of a muscle to produce force in any objective, measurable context. Throughout the physical therapy and rehabilitation literature, sEMG amplitude thresholds for adaptation have been suggested; for example, thresholds of 40–60% of MVIC have been suggested to be necessary for strength gains to be elicited from a given muscle (Andersen et al., 2006; Ayotte et al., 2007; Escamilla et al., 2010; Reiman et al., 2012). It seems that these recommendations for sEMG interpretation stem from training load recommendations (Andersen et al., 2006). The rationale behind this is simply that sEMG amplitudes increase, or are correlated, with force, and greater loads are needed for greater strength gains. Implicated by such a rationale are a couple of assumptions that will be addressed: (1) normalized sEMG amplitude is equal to the relative load of an exercise, and (2) the relative loads provided are actually what is needed to improve strength.

The supposition that sEMG amplitude is equal to the relative load of an exercise *may* be true for some isometric muscle actions when performed in the same position to which sEMG has been normalized (Alkner et al., 2000), although not always (see section Acute and Mechanistic Variables) (Potvin et al., 1996). However, in the context of dynamic exercises, or when positions differ from the normalization position, this relationship does not hold (Aspe and Swinton, 2014; Calatayud et al., 2015; Vigotsky et al., 2015). Accordingly, there is no basis to assume that loading recommendations can be extrapolated to sEMG amplitude recommendations.

The second assumption of this rationale is that heavier loads are needed to increase strength. Indeed, while this is in line with the principle of specificity, in some contexts, lighter loads (<40%) have also been shown to have the ability to increase strength (Mitchell et al., 2012; Morton et al., 2016), albeit not as well as heavier loads (Schoenfeld et al., 2015). Moreover, there are data to suggest that strength gains may occur from unloaded activities, such as downhill walking (Maeo et al., 2015, 2016), which only elicits EMG amplitudes of ~30% MVIC for the vastus medialis and rectus femoris (Maeo et al., 2012). The construct of strength gain is not straightforward, as results will depend on how strength is tested (Buckner et al., 2016; Gentil et al., 2017) and that strength gains are not a binary outcome, but rather, they occur on a continuum. As such, we feel that this premise is not supported by more recent literature. Because strength is born from a variety of exercise programming variables (load, frequency, volume, etc.) and characteristics (kinetics and kinematics), more work is needed to understand exactly how sEMG amplitudes play an interactive role in exercise prescription. Finally, because strength is often tested as an emergent property of one or multiple joints, adaptations of surrounding musculature (agonists, synergists, and antagonists) must also be considered; these adaptations may decrease the relative importance of single-muscle adaptation for single- or multi-joint strength outcomes (de Boer et al., 2007).

An interesting and relevant longitudinal study was carried out by Calatayud et al. (2015), who had participants perform either a 6 RM elastic band-resisted push-up or 6 RM bench press over a 5-week period. Acutely, these exercises elicited similar sEMG amplitudes of the pectoralis major and anterior deltoid, and longitudinally, resulted in similar increases in bench press 6 RM and 1 RM (Calatayud et al., 2015). While this is a step in the right direction, the similarities in sEMG amplitude may very well have been an epiphenomenon rather than a cause for the strength outcomes. That is, both sEMG amplitudes and strength gains may have been a function of training load. More studies are needed that manipulate the relationship between load and sEMG amplitude to filter out their relative importance in rendering strength gains.

#### Interim summary

Muscular strength and hypertrophy are adaptations of interest for practitioners of all types, ranging from physical therapists to trainers and coaches. Due to the ease-of-use of sEMG and the seemingly logical basis for inferring strength and hypertrophy adaptations from sEMG amplitude, numerous authors have attempted to utilize sEMG amplitude as a surrogate endpoint for these longitudinal measures. Doing so, however, is not supported by the current body of literature. We strongly urge practitioners and researchers to view acute data through a critical lens; implying and inferring longitudinal outcomes from acute data should be condemned until acute measures have been validated as surrogate outcomes (Halperin et al., 2017). Lastly, from some of the data presented hitherto, it is likely that sEMG amplitudes will be interactive with other exercise programming variables in the determination of longitudinal adaptation. This is not a limitation of sEMG, but rather a limitation of working with complex biological systems.

## What conclusions *can* be drawn from sEMG studies (with caution)?

Up to this point, the limitations of sEMG have been covered in great detail. However, it is prudent to discuss how sEMG can be properly applied and interpreted, in addition to how gray areas can affect interpretation. Therefore, this section focuses on what conclusions can “cleanly” be drawn from sEMG and under what conditions one must approach sEMG interpretations with more caution.

### Binary excitation (on/off)

**Figure 4:**
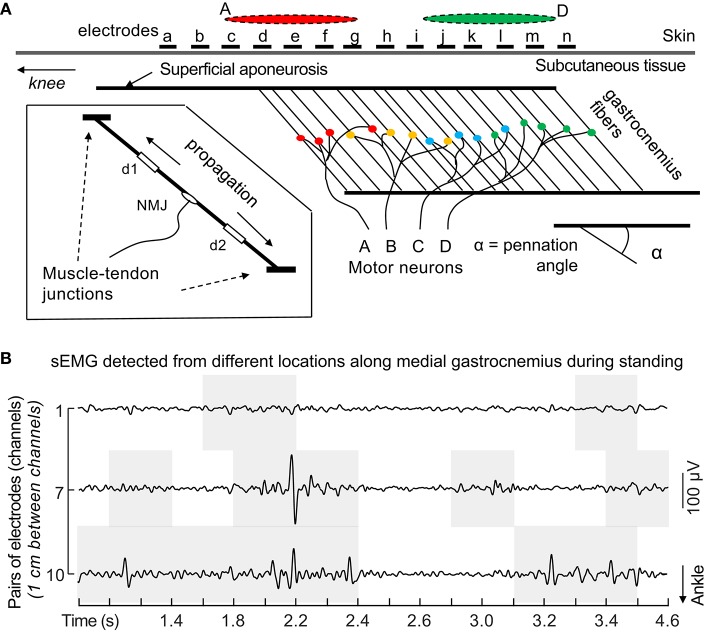
Under-representative sampling of motor units with sEMG. **(A)** Unlike observed for skin parallel-fibered muscles, the same action potentials propagating along the fibers of pennated muscles are not sampled by a single pair of surface electrodes positioned anywhere on the muscle. As exemplified above, action potentials of motor units A and D are detected mainly by proximal and distal electrodes (red and green ellipses), respectively. sEMG amplitudes detected locally likely provide an unrepresentative view of the actual degree of muscle excitation. Biased inferences may be drawn from unrepresentative sEMG. Consider, for example, the amplitude of sEMG detected by any pair of distal electrodes in the figure (from *i* to *n*). If, during a given submaximal contraction, only the distal muscle region is excited, normalized sEMG amplitude will likely indicate a nearly 100% degree of excitation. Conversely, normalized sEMG detected proximally would overly underestimate the degree of excitation. These considerations presume excitation of the whole muscle volume during the reference, normalization condition. Inferences on the degree and timing of excitation are not supported by EMGs detected locally from large, pennated muscles. Reproduced from Merletti et al. (2016), with permission. **(B)** Raw sEMG detected with pairs of electrodes from different locations along medial gastrocnemius. Gray, shaded areas indicate periods when the root mean square amplitude (30 ms epochs) was greater than the background, rest level during a standing task. *Nota bene* the false conclusions that can be drawn regarding the state of a muscle by looking at only one pair of electrodes (e.g., electrode pair #1). Reproduced from dos Anjos et al. (2017) (under CC-BY 4.0).

Knowing the state of a muscle is incredibly important for experiments that aim to measure variables that require no active contributions; for example, passive stiffness. This is often thought to be one of the most valid applications of sEMG, provided that the electrode is representative of the entire muscle (Cavanagh, 1974; Farina, 2006). This, however, is not always the case, as electrodes only cover a small, select portion of muscle (Figure 4A). Indeed, recent evidence suggests that some parts of a muscle may be electromyographically “silent” while other parts are not (dos Anjos et al., 2017; Figure 4B). These uncertainties likely also apply to greater contraction intensities, as *negative* electromechanical delay was recently observed in up to 23% of isometric elbow flexion trials with net joint moments ranging from 20 to 70% MVIC (Dieterich et al., 2017). These data suggest that an electrode, for a short period of time, is not representative of the entire muscle over which it is placed (Dieterich et al., 2017). It has been proposed that such findings are at least partially a function of interelectrode distance; smaller interelectrode distances result in smaller pick-up volumes, which decreases how representative a sEMG signal is of the muscle over which the electrodes are placed (Vieira et al., 2017). Fortunately, the periods during which electrodes are poorly representative of a muscle's binary state (on/off) are often transient (Figure 4B), and thus, it is likely that an electrode will provide insight as to the muscle's current state *eventually*. In other words, although an electrode may not accurately represent the state of a muscle at any instant (a single time point), it will provide the state of the muscle over longer periods of time. When dealing with short bursts of excitation, the implications are less clear and more caution should be used when interpreting results (Cavanagh, 1974). It follows that one may reasonably conclude whether or not a muscle is “on” or “off” if the time of interest is >~200 ms (Dieterich et al., 2017). Finally, one can increase their confidence in knowing the state of a muscle by using multiple electrodes or HD-sEMG, which accounts for intramuscular heterogeneity (Dieterich et al., 2017; dos Anjos et al., 2017; Le Mansec et al., 2017).

### Timing of excitation

Much like knowing if a muscle is “on” or “off,” it can be incredibly insightful to know *when* a muscle changes its state; for example, determining *when* a muscle turns “on” during the gait cycle. Such insight has applications for musculoskeletal modeling, understanding pathology, and basic motor control. Due to the variability in electromechanical delays (Dieterich et al., 2017), gathering the timing of activation can be more complicated (Cavanagh, 1974). Thus, depending on the application and speed of movement (e.g., isometric vs. ballistic), electromechanical delays—although on the order of a few tenths of a second—may need to be considered (Cavanagh, 1974), as should the possibility that an electrode may not readily represent the state of a muscle (dos Anjos et al., 2017; Figure 4). Notwithstanding these points, if small errors are acceptable for the application in question (e.g., <200 ms; Dieterich et al., 2017), then sEMG can be very useful for this purpose. Finally, one can increase their confidence in knowing the state of the muscle by using multiple electrodes or HD-sEMG (Figure 4; Dieterich et al., 2017; dos Anjos et al., 2017; Le Mansec et al., 2017).

### Relative muscle excitation

Muscle excitation is the process by which a motor neuron action potential depolarizes the sarcolemma of muscle fibers. This process leads to muscle activation and then the production of muscle force (Figure 1). Because muscle excitation involves the depolarization of a muscle fiber (muscle fiber action potential), the sum of muscle excitations can be recorded using sEMG (Figure 3C). Thus, rectified sEMG amplitude scales with the level of global muscle excitation (Figure 3C). However, when attempting to compare excitation between timepoints, individuals, or muscles, a number of factors must be considered. The following subsections describe considerations when attempting to draw conclusions on relative muscle excitation, and what information can be obtained under “perfect” experimental conditions, with controls for important confounding variables (e.g., electrode movement and changes in tissue conductivity) (Farina, 2006). It should be noted that such factors are quite difficult to control during dynamic contractions; in many cases, these factors are inherent and thus are impossible to control for (Farina, 2006). A summary of this section is shown in Table 1, and all of the examples/applications provided assume the consideration criteria provided in Table 1.

**Table 1:** Examples of practical research questions and possible conclusions that can be drawn based on the relative excitation of muscle from sEMG amplitudes.

**Within-muscle**	**Within-subject**	**Between-subjects**
Acute	Changes in neural drive, potentially associated with changes in motor performance. Does the decrease in force output following stretching stem from changes in neural drive? Trajano et al., 2013 Criteria 1–3, 6, 7, and sometimes 8	Can inform potential mechanisms for differences in function. Do stronger individuals have greater muscle excitation? Trezise et al., 2016 Criteria 1–6, 7, and sometimes 8
Longitudinal	Can explain how changes in function occur. Does agonist muscle excitation change following a resistance training program? Moritani and deVries, 1979 Criteria 1–7 and sometimes 8	Can explain differential mechanisms for changes in function. Do different resistance training interventions lead to (dis)similar muscle excitation, and are those changes related to differences in strength gain? Jenkins et al., 2017 Criteria 1–7 and sometimes 8
**Between-muscles**	**Within-subject**	**Between-subjects**
Acute	Provide insight into how changes in function may arise from acute interventions. Are differences in co-contraction with different instructions associated with different strength measures? Lohse et al., 2011 Gain a better understanding of which, and to what extent, different muscles are recruited during an exercise. Santana et al., 2007 Criteria 1–3, 5, 7, 8, and 9	When used with statistical analysis, can provide information about complex neuromuscular pathology How does pathology affect motor control? Steele et al., 2015 Can inform mechanisms for differences in function. Do those who exhibit greater strength have less co-contraction? Macaluso et al., 2002 Criteria 1–6, 7, and 8
Longitudinal	Can help explain how changes in function arise. Is the reduction in co-contraction following resistance training associated with increased strength? Erskine et al., 2010, 2014 Criteria 1–9	Can help explain how changes in function arise. Can divergent changes in strength following an intervention be explained by changes in co-contraction? Erskine et al., 2010, 2014 Criteria 1–9

*Examples of valid sEMG questions from the literature are described, along with criteria that must be fulfilled in order to draw valid conclusions from those studies. Note that the referenced studies do not necessarily fulfill the criteria*.

*Operational definitions:*
Function = proficiency in performing a motor task; Acute = within the same session, electrodes are not removed; Longitudinal = measurements are made before and following an intervention or over time, electrodes are removed and reapplied, usually occurs over weeks or months; Within-muscle = all comparisons are made within the same muscle (e.g., medial gastrocnemius vs. medial gastrocnemius); Between-muscle = comparison requires measurements from at least two muscles (e.g., a ratio between two muscles or directly comparing EMG amplitudes of different muscles).

*The condition criteria are as follows:*
Electrodes are not positioned symmetrically with regard to the muscle innervation zone; Negligible crosstalk from nearby muscles; Electrodes sample from most of the whole volume of the target muscles (i.e., electrode is representative of the muscle); Electrodes are positioned at the same skin location relative to the muscle; Anatomical changes/differences (subcutaneous tissue, etc.) are controlled for; Minimal changes/differences in biochemical environments; Kinematics (i.e., position/ROM and velocity) are kept constant between trials, subjects, and/or muscles that are being compared; Signals are normalized, preferably to maximum M-wave amplitude; and *Recruitment characteristics (deep-to-superficial, superficial-to-deep) must be considered*.

#### Within-subject, within-muscle

##### Considerations

When performed acutely, within a single session, relative muscle excitation can be measured, assuming that fatigue, electrode position/movement (relative to both the skin and underlying motor units), tissue conductivity, and signal nonstationarity (rapid changes in signal properties) are taken into account (Farina, 2006). Longitudinally, especially following a training program, such results should be more strongly scrutinized (Arabadzhiev et al., 2014). In such cases, changes in sEMG amplitude may arise from not only central factors^4^, but also peripheral factors^5^. Because these changes may arise from *both* neural *and* peripheral sources, it can be difficult to draw meaningful conclusions. In many cases, central adaptations may not be able to be discerned from peripheral adaptations using just sEMG.

##### Applications

Perhaps one of the most common types of comparisons made in sport and rehabilitation sciences are within-subject, within-muscle comparisons. For example, researchers will compare sEMG amplitudes elicited during different exercises (Andersen et al., 2006; Escamilla et al., 2010). Notwithstanding the noted considerations, acute within-subject, within-muscle (i.e., pre- and post-conditions within the same session) studies performed under controlled conditions (i.e., kinematically-matched; preferably isometric) may provide useful information. For example, Trajano et al. (2013) used sEMG normalized to maximum M-wave amplitude to help tease out peripheral factors from central factors that affect force loss following an acute bout of static stretching (Table 1). Longitudinally, sEMG may inform mechanisms of changes in function, so long as the aforementioned potential confounders are controlled for or, at the very least, taken into consideration (Table 1).

#### Within-subject, between-muscles

##### Considerations

For measures to be indicative of muscle excitation, the previously-discussed confounding variables (fatigue, electrode movement, tissue conductivity, and nonstationarity) must be taken into account; tissue conductivity will necessarily be different between muscles, due to differences in geometry and dynamics (Farina, 2006). It should also be considered that an individual may be better able to voluntarily excite one muscle more than another during an MVIC task, or alternatively, electrodes may be more or less representative of a respective muscle (Figure 4). Both of these scenarios may result in altered (inflated or deflated) normalized sEMG amplitudes^6^. Moreover, the kinematics of the muscle over which the electrode is placed must be considered: Are the relative lengths and velocities of the muscles in question comparable, and how is electrode position relative to each muscle's innervation zone changing? One should be wary when attempting to compare excitation patterns of different muscles, especially during dynamic efforts (Farina, 2006).

##### Applications

If signals are properly normalized and appropriate considerations are made (Table 1), one may infer relative muscle excitation from within-subject, between-muscle sEMG studies. For example, such studies may provide insight as to how differences in strength arise following verbal cueing (Lohse et al., 2011) or how co-contraction following a resistance training program may help explain changes in strength (Erskine et al., 2010, 2014; Table 1). Other studies have utilized sEMG to compare which muscles experience the greatest excitation during different exercises (Santana et al., 2007; Table 1), but interpreting what these differences mean is ambiguous (see section What Conclusions Cannot Be Drawn from sEMG Studies?).

#### Between-subjects, within-muscle

##### Considerations

Much like comparing between-muscles, there are a number of concerns pertaining to whether or not individuals in a group, especially symptomatic ones, have the ability to maximally excite a muscle during a normalization trial^7^. Furthermore, different participants respond differently to different normalization positions and techniques (Vera-Garcia et al., 2010; Contreras et al., 2015), so one can never be sure whether they are comparing apples-to-apples, so to speak, unless more robust normalization techniques are used, such as maximum M-wave amplitude. In such cases, training (exercise) experience may still confound maximum M-wave normalized sEMG signals (Arabadzhiev et al., 2014). Therefore, comparisons between subjects of starkly different populations (trained vs. untrained or those in pain vs. not in pain) may be confounded (Farina, 2006).

##### Applications

In more homogeneous populations, one can use sEMG to understand mechanisms for differences in function. For example, authors have used maximum M-wave normalized sEMG amplitudes to help us understand differences in strength between individuals (Trezise et al., 2016) and to explain how changes in strength may arise following different interventions (Jenkins et al., 2017; Table 1).

#### Between-subjects, between-muscles

##### Considerations

The considerations for between-subject and between-muscle comparisons above both apply to this category, but are amplified due to the larger potential for differences to exist.

##### Applications

sEMG can be useful for comparing co-contraction between populations—such young and old individuals—to understand how differences in function—such as strength—may arise (Macaluso et al., 2002; Table 1). Moreover, sEMG from several muscles drive muscle synergy analyses, which are showing to be informative for understanding neural control differences in those with cerebral palsy or changes following a stroke (Steele et al., 2015; Table 1). Muscle synergy analyses can also be applied to experimental questions in sport, which could provide insight into motor control strategy differences between populations while carrying out a task (e.g., expert vs. non-expert) or under different biomechanical constraints (e.g., limiting degrees of freedom or adding external load). Longitudinally, one may wish to understand how between-muscle neural control strategies explain differential adaptations to an intervention (Erskine et al., 2010, 2014; Table 1).

## How can sEMG be made more applicable?

Challenging the applicability of sEMG can be viewed as bittersweet. While it is quite humbling that we do not truly understand what we are measuring or its implications, this also opens the door for high quality, impactful research. Ultimately, longitudinal outcomes are likely of greatest interest to practitioners (Halperin et al., 2017). Therefore, there is a tremendous need to evaluate the ability of sEMG to be used as a surrogate endpoint for muscle strengthening and hypertrophy (Halperin et al., 2017). We, the authors, have pondered potential study designs to investigate this question but have been unable to ideate something that we believe is sufficient to answer the question. While previous investigations may be thinking along the right lines, they do not provide robust evidence (Calatayud et al., 2015). However, we encourage readers and fellow scientists to brainstorm and carry out such research, with the end goal of determining (1) if sEMG amplitude can be predictive of strength improvements or hypertrophy, (2) what the minimum difference in sEMG amplitude is for predicting greater strength or hypertrophy adaptations, (3) how generalizable the results are, and (4) how new technologies and more advanced signal processing techniques can be utilized. Indeed, hypertrophy and strength are likely highly multifactorial and nonlinear, which will make such research tremendously difficult, if not impossible. Similar work has already been carried out as it pertains to muscle protein synthesis and hormone responses to exercise bouts (Mitchell et al., 2012, 2014; West and Phillips, 2012; Damas et al., 2016; Morton et al., 2016), and we believe it is time that sEMG be scrutinized in a similar fashion. Such research will validate or invalidate hundreds, if not thousands, of sEMG studies that were intended to be extrapolated for these longitudinal outcomes.

## Summary of considerations for the applicability of sEMG

1. Factors other than muscular effort influence the myoelectric signal, including muscle length, contraction mode, contraction speed, etc. Comparing sEMG signals between different exercises that do not control for these variables should be avoided.
2. Even when the sEMG signal adequately represents the force of the muscle, caution should be exercised when concluding that a certain exercise will be better for increasing strength or hypertrophy due to other factors that influence these adaptations.
3. Comparing normalized sEMG values between individuals with and without pain should be viewed cautiously. Changes in the normalized sEMG value over time cannot indicate changes in excitation because the normalized value can be influenced by excitation during the normalization contraction or during the measured exercise. Normalizing to maximum M-wave amplitude can, to an extent, help diminish such effects.
4. Within-subject, within-muscle comparisons of the sEMG signal across different exercises may be able to provide insight into muscular force production, provided the previously mentioned controls are made.

## Conclusions

sEMG is a useful tool for gaining insight into the neuromuscular system, musculoskeletal modeling, and basic science work, but its practical applicability is limited at present. Researchers wishing to produce applicable research pertaining to longitudinal adaptation should prioritize longitudinal studies rather than acute, cross-sectional sEMG work (Halperin et al., 2017). Because sEMG has not been validated as a surrogate endpoint for longitudinal measures, readers should be wary of bold conclusions. Important mechanistic details of sEMG, such as signals being confounded by peripheral factors and data not being representative of a muscle, must be considered when attempting to draw conclusions—even acute, mechanistic ones. For these points, we wish to stress that the burden of proof is on researchers to show that cross-sectional sEMG findings are practically meaningful for longitudinal outcomes, and until this is shown, discussions and conclusions should not imply that they are. Finally, although this review was expansive, depth was sacrificed for breadth and communicabilty. For readers interested in learning more about some of the topics discussed in this review, recommended texts, chapters, papers, and reviews are provided in Table 2.

**Table 2:** Further reading.

**Topic**	**References**
Basic biophysics of sEMG	Enoka, 2015; Farina et al., 2016
sEMG during fatiguing contractions	Dimitrova and Dimitrov, 2003; Arabadzhiev et al., 2010b; Potvin and Fuglevand, 2017
sEMG during dynamic contractions	Farina, 2006
sEMG and muscle force	Staudenmann et al., 2010
Muscle recruitment characteristics	De Luca and Kline, 2012
sEMG limitations and interpretations	Cavanagh, 1974; Clarys and Cabri, 1993; De Luca, 1997; Clarys, 2000; Soderberg and Knutson, 2000; Pieter Clarys et al., 2010; Enoka and Duchateau, 2015
Reporting standards	Winter et al., 1980; Hermens et al., 1999; ISEK, 2017

## Author contributions

AV, IH, GT, and TV conceived the manuscript. AV, IH, GL, GT, and TV wrote, reviewed, and approved the final version of the manuscript. AV and TV created the figures. AV and IH constructed the tables.

### Conflict of interest statement

The authors declare that the research was conducted in the absence of any commercial or financial relationships that could be construed as a potential conflict of interest.

## Footnotes

^1^This is not representative of the number of active cross-bridges in the muscle, given the number of binding sites in an active muscle changes with its length. For example, the number of cross-bridges established in an active muscle reduces as the muscle shortens from its optimal length. Therefore, such measurements are *not* a measure of muscle force, namely due to contraction dynamics.

^2^It should be noted that both of these mechanisms only pertain to *active* force production, due to cross-bridging, and are independent of passive contributors to force production (e.g., parallel elastic elements).

^3^Units are arbitrary, relative to the number of active fibers and the muscle fibers' optimal length, respectively.

^4^Central factors include, but are not limited to, corticomotor (Fisher et al., 2016) and motoneuron (Heckman et al., 2009) excitability.

^5^Peripheral factors include, but are not limited to, increased intracellular action potentials due to changes in the biochemical environment (e.g., increased resting calcium levels, Arabadzhiev et al., 2014), changes in tissue properties (e.g., subcutaneous fat, Kuiken et al., 2003), and changes in muscle architecture (Lieber and Fridén, 2000).

^6^Normalizing to a submaximum level of excitation when maximum is assumed will inflate the normalized sEMG amplitude. This effect may be amplified with differential recruitment patterns (Figure 2). Moreover, in the case of submaximal normalizations, the linear assumption of normalizations may be violated (Hug and Tucker, 2017).

^7^Pain centrally inhibits muscle excitation (Farina et al., 2005) and changes EMG-to-force ratios (Graven-Nielsen et al., 1997) without affecting muscle function (Arvidsson et al., 1986; Graven-Nielsen et al., 1997). Thus, someone in pain may produce lower excitation during a normalization trial (e.g., MVIC), which would inflate normalized sEMG amplitudes.
